# A scFv antibody targeting common oligomeric epitope has potential for treating several amyloidoses

**DOI:** 10.1038/srep36631

**Published:** 2016-11-08

**Authors:** Jun Zha, Xiang-meng Liu, Jie Zhu, Shu-ying Liu, Shuai Lu, Peng-xin Xu, Xiao-lin Yu, Rui-tian Liu

**Affiliations:** 1 State Key Laboratory of Biochemical Engineering, Institute of Process Engineering, Chinese Academy of Sciences, Beijing, China; 2 School of Pharmacy, China Pharmaceutical University, Nanjing, China; 3 School of Bioengineering, Qilu University of Technology, Jinan, China; 4 School of Life Science, Ningxia University, Yinchuan, China

## Abstract

Overproduction or poor clearance of amyloids lead to amyloid aggregation and even amyloidosis development. Different amyloids may interact synergistically to promote their aggregation and accelerate pathology in amyloidoses. Amyloid oligomers assembled from different amyloids share common structures and epitopes, and are considered the most toxic species in the pathologic processes of amyloidoses, which suggests that an agent targeting the common epitope of toxic oligomers could provide benefit to several amyloidoses. In this study, we firstly showed that an oligomer-specific single-chain variable fragment antibody, W20 simultaneously improved motor and cognitive function in Parkinson’s disease and Huntington’s disease mouse models, and attenuated a number of neuropathological features by reducing α-synuclein and mutant huntingtin protein aggregate load and preventing synaptic degeneration. Neuroinflammation and oxidative stress *in vivo* were also markedly inhibited. The proposed strategy targeting the common epitopes of amyloid oligomers presents promising potential for treating Parkinson’s disease, Huntington’s disease, Alzheimer’s disease, and other amyloidoses.

Amyloid protein misfolding and pathological aggregation are considered common hallmarks of amyloidoses^1^,^2^. More than 40 different diseases, such as Alzheimer’s disease (AD), Parkinson’s disease (PD), and Huntington’s disease (HD), have been associated with amyloid aggregation, and some of these diseases cause great social and economic burdens because of their extensive prevalence and lack of effective therapy^3^,^4^. Amyloid aggregation processes can be initiated by overproduction or poor clearance of disease-related proteins. Amyloid monomers undergo conformational changes, resulting in misfolding, aggregating into small oligomers and protofibrils, or finally stabilizing as mature fibrils^4^. Numerous clinical observations have confirmed that the severity of amyloid-associated neurodegenerative diseases was not correlated with the amount of amyloid deposit in the brain of patients, but was correlated with elevated levels of toxic oligomers^5^,^6^. Various studies have revealed that amyloid oligomers, rather than monomers or insoluble fibrils, are the primary toxic species in the pathological processes of amyloidoses^7^,^8^. The mechanisms of toxicity of the oligomers are they interact with the lipid bilayer of the cell membranes, leading to membrane disruption or even pore formation, inducing oxidative stress by generation of reactive oxygen species (ROS), in turn causing lipid and protein oxidation, mitochondrial dysfunction, disturbance of autophagy and changes in ion homeostasis, and cell death eventually^9^,^10^.

Different amyloids have distinct amino acid sequences, but their oligomers contain common structures with rich cross-β-sheets and share universal mechanisms of toxicity^11^,^12^. Thus, amyloid oligomers can be considered perfect therapeutic targets. During one amyloid aggregation and amyloidosis development, other amyloids may be induced to aggregate and participate in the pathological processes of amyloidoses. Emerging evidence showed that Aβ, tau, and α-synuclein may interact synergistically to promote their aggregation and accumulation and accelerate neuropathology and cognitive dysfunction^13^,^14^. Moreover, amylin was found to co-precipitate with Aβ to form complex amylin/Aβ plaques in the brains of AD patients^15^, and mature amylin fibrils promoted the robust growth of mixed amylin/Aβ amyloids^16^. Furthermore, oligomers of α-synuclein, prion protein, TDP-43, tau, and Aβ, were detected in the brains of AD patients^17^,^18^,^19^,^20^, suggesting that direct treatment of one amyloid protein may not be sufficient to cure the disease^21^. Thus, developing agents that target common structures of oligomers assembled from different amyloids could be a promising strategy to treat one amyloidosis or several amyloidoses.

PD is the second most frequent neurodegenerative disease in humans, which is characterized pathologically by the formation of intraneuronal inclusions called Lewy bodies. α-synuclein aggregates are the main components of Lewy bodies, and they induce the progressive death of dopamine-producing neurons in the substantia nigra of the midbrain, leading to motor and cognitive deficits^22^. HD is another fatal neurodegenerative disorder characterized by progressive motor, cognitive, and psychiatric deficits, which is due to the aggregates of mutant huntingtin protein (mHTT) composed of an extended polyglutamine (polyQ) tract^23^. mHTT oligomers are the main neurotoxic factors that induce the death of striatal and cortical neurons^8^. Thus, inhibiting the aggregation and cytotoxicity of α-synuclein and mHTT is a potential approach for treating PD and HD.

Some anti-oligomer antibodies, such as A11, NU4, and F11G3, have significantly improved cognitive impairment in AD transgenic mice^24^,^25^, but few agents have been reported to simultaneously exert beneficial effects on AD, PD, and HD animal models. We previously reported a conformation-dependent oligomer-specific single-chain variable fragment (scFv) antibody W20, which was isolated from a naïve human scFv library via phage display, recognized various oligomers assembled from Aβ, α-synuclein, amylin, insulin, prion protein, lysozyme and polyQ. W20 also inhibited the fibrillation of numerous amyloids and attenuated amyloid oligomer-induced cytotoxicity *in vitro*^26^,^27^. In another work, W20 ameliorated memory deficits and brain amyloid burden in AD transgenic mice^28^. Considering the pathological processes of one amyloidosis may be simultaneously associated with several types of amyloids, and W20 is a pan-amyloid oligomer specific antibody, in the present study, we separately investigated the effect of W20 on the behavior, cognition, and neuropathology in both PD and HD transgenic mouse models to explore whether W20 is suitable for treating different amyloidoses.

## Results

### W20 improved motor performance in A53T α-synuclein mice

**Figure 1:**
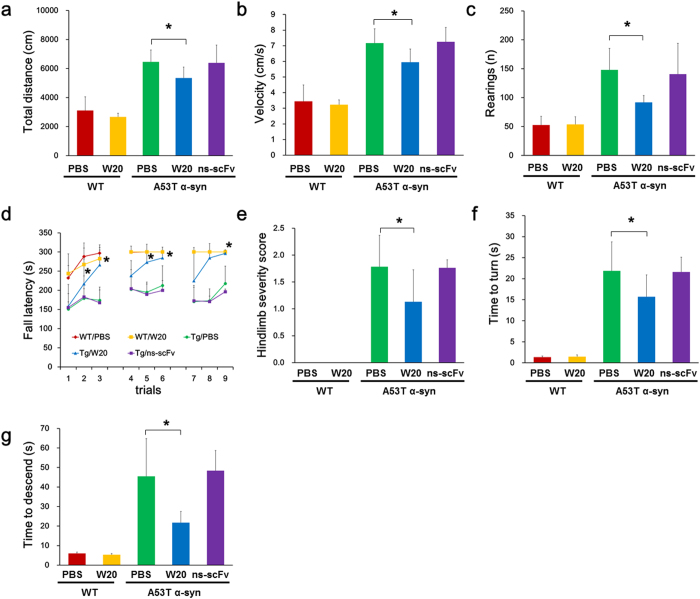
W20 improved motor performance in A53T α-synuclein mice. (**a–c**) The locomotion behavior of vehicle or W20-treated WT and A53T α-synuclein mice was assessed by open field test. Total distance traveled (**a**), mean velocity during the exploration period (**b**), and rearing frequency (**c**) were measured. *n* = 8 mice/group. *P < 0.05, one-way ANOVA followed by Tukey’s *post hoc* test. (**d**) Rotarod tests were performed in nine accelerating rotarod trials over 3 consecutive days. The average latency to fall was determined. *n* = 8 mice/group. *P < 0.05, one-way ANOVA followed by Tukey’s *post hoc* test. (**e**) Hindlimb clasping behavior was assessed. The hindlimb clasping score was rated from 0-3 based on severity. *n* = 8 mice/group. *P < 0.05, one-way ANOVA followed by Tukey’s *post hoc* test. (**f,g**) Motor coordination were measured in pole test by the time to its head downwards (**f**) and time to descend (**g**). *n* = 8 mice/group. *P < 0.05, one-way ANOVA followed by Tukey’s *post hoc* test. Data represent means ± SD.

To assess the effect of W20 on locomotion and reaction to a novel environment in PD animal model, we performed open field test on transgenic A53T α-synuclein mice, which overexpressed A53T mutant human α-synuclein under the mouse prion promoter and developed severe motor impairment and cognitive deficits by accumulation of A53T α-synuclein at 9–16 months of age^29^. The results showed that A53T α-synuclein mice treated with the vehicle or non-specific (ns) - scFv travelled a longer distance (Fig. 1a), exhibited higher movement velocity (Fig. 1b), and reared more frequently (Fig. 1c) than wild-type (WT) mice. These findings demonstrated that A53T α-synuclein mice were hyperactive, which was consistent with the previous reports^30^,^31^. By contrast, W20-treated A53T α-synuclein mice exhibited decreased locomotion, velocity, and rearing compared with vehicle-treated mice (Fig. 1a–c).

In addition to the alterations in locomotion, A53T α-synuclein mice also displayed progressive motor function impairment by falling off the rotarod sooner than WT mice treated with either the vehicle or W20 in all trials, whereas W20-treated A53T α-synuclein mice stayed on the accelerating rotarod longer than vehicle- or ns-scFv-treated controls and showed significant improvement in rotarod latency (Fig. 1d).

Hindlimb clasping has been observed in various neurodegenerative mouse models. A53T α-synuclein mice exhibited severe hindlimb clasping behavior compared with the WT littermates. W20 treatment improved this behavior as illustrated by the remarkable decrease in hindlimb severity score (Fig. 1e).

The pole test was utilized to measure motor coordination and balance in mouse models of PD. A53T α-synuclein mice treated with the vehicle or ns-scFv took a longer time to turn (Fig. 1f) and descend from the pole (Fig. 1g) compared with the WT littermates. By contrast, W20 substantially improved the deficits in motor coordination of A53T α-synuclein mice by reducing movement initiation and descending time in the pole test. These results suggested that W20 significantly restored motor behavior in A53T α-synuclein mice.

### W20 attenuated cognitive deficits in A53T α-synuclein mice

**Figure 2:**
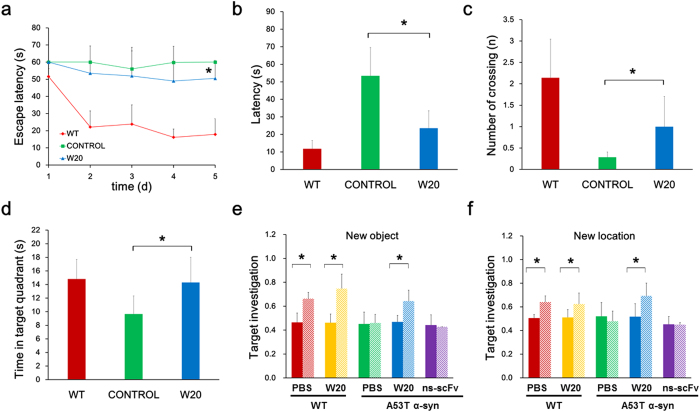
W20 attenuated cognitive deficits in A53T α-synuclein mice. (**a–d**) Spatial learning and memory retention of WT and A53T α-synuclein mice were assessed using the Morris water maze after treatment with W20 or vehicle. (**a**) During training trials, the latency to find the hidden platform were measured. (**b–d**) During probe trials, the latency to the position of the removed platform (**b**), the number of platform crossings (**c**), and the time spent in the target quadrant (**d**) were measured. *n* = 8 mice/group. *P < 0.05, one-way ANOVA followed by Tukey’s *post hoc* test. (**e,f**) Object recognition was performed to test the cognition of mice treated with or without W20 by the novel object preference (**e**) and novel object location assays (**f**). *n* = 8 mice/group. *P < 0.05, one-way ANOVA followed by Tukey’s *post hoc* test. Data represent means ± SD.

The Morris water maze (MWM) test was conducted to assess spatial learning and memory in A53T α-synuclein mice. During the acquisition period, both WT and W20-treated A53T α-synuclein mice required shorter time to reach the platform after training compared with vehicle-treated A53T α-synuclein mice (Fig. 2a). During the memory retention test in the probe trials, W20-treated mice exhibited spatially oriented swimming behavior and shorter escape latencies than vehicle-treated mice (Fig. 2b). Moreover, W20-treated A53T α-synuclein mice showed higher frequency of platform crossings (Fig. 2c) and spent more time in the target quadrant (Fig. 2d) compared with vehicle-treated mice. These results suggested that A53T α-synuclein mice exhibited impaired spatial learning and memory, whereas W20 treatment significantly improved the cognitive impairment of the PD mouse model. The spatial memory of the mice was further evaluated with the object recognition test. WT mice preferred to investigate the target object and exhibited a significant increase in the percentage of investigations to the target object during training and test session (Fig. 2e,f). A53T α-synuclein mice did not show a preference for the target object. However, W20-treated A53T α-synuclein mice displayed improved object recognition by showing remarkable preference to investigate both a novel object (Fig. 2e) or a known object at a novel location (Fig. 2f). These results further indicated that W20 treatment reversed the memory deficits in A53T α-synuclein mice.

### W20 reduced α-synuclein levels in A53T α-synuclein mouse brains

**Figure 3:**
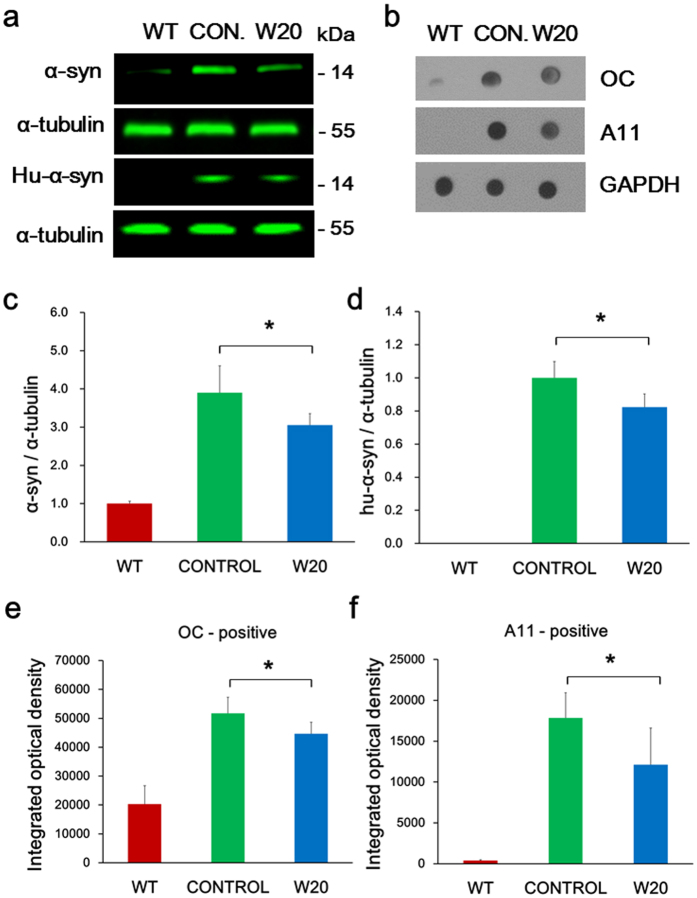
W20 reduced α-synuclein levels in A53T α-synuclein mouse brains. (**a**) Western blot was performed to measure total α-synuclein and overexpressed human A53T α-synuclein levels in WT and A53T α-synuclein mouse brain lysates. α-synuclein protein signal intensity for each sample was normalized to α-tubulin protein signal intensity. (**b**) Dot-blot was conducted to measure both OC-positive fibrillar oligomers (FOs) and A11-positive prefibrillar oligomers (PFOs) levels in WT and A53T α-synuclein mouse brain lysates. Oligomer signal intensity for each sample was normalized to GAPDH signal intensity. Total α-synuclein (**c**) and overexpressed human A53T α-synuclein levels (**d**) in WT and A53T α-synuclein mouse brain lysates were quantitatively analyzed. OC-positive FOs (**e**) and A11-positive PFOs (**f**) levels in WT and A53T α-synuclein mouse brain lysates were quantitatively analyzed. *n* = 6 mice/group. *P < 0.05, Student’s *t*-test. Data represent means ± SD.

The amount of α-synuclein in the brains of A53T α-synuclein mice are correlated with disease onset and mouse survival^29^. To determine whether the motor and memory improvements by W20 treatment were associated with the reduction of α-synuclein levels, we performed western blot using mice brain lysates. Levels of α-synuclein in the brain lysates of A53T α-synuclein mice were obviously higher compared with those of the WT littermates (Fig. 3a). However, W20 treatment significantly decreased levels of total α-synuclein (Fig. 3a,c) and overexpressed human A53T α-synuclein (Fig. 3a,d) levels, as detected by Syn-1 and LB509 antibody, respectively. It is notable that ns-scFv, unlike W20, did not affect the levels of α-synuclein in the brains of A53T α-synuclein mice. Moreover, both PBS and W20 did not affect the levels of α-synuclein in WT mice, suggesting that W20, but not ns-scFv had specific therapeutic function on PD mice (Supplementary Fig. 1). Soluble α-synuclein oligomers are considered the most neurotoxic forms in PD. We determined the levels of α-synuclein oligomers in the brain lysates by dot-blot using oligomer-specific antibodies OC and A11 (Fig. 3b). W20-treated mice showed 13.6% (Fig. 3e) and 32.1% (Fig. 3f) reductions in levels of α-synuclein oligomers, as detected by OC and A11 antibodies, respectively, compared with vehicle-treated A53T α-synuclein mice.

### W20 attenuated neuropathology in A53T α-synuclein mice

**Figure 4:**
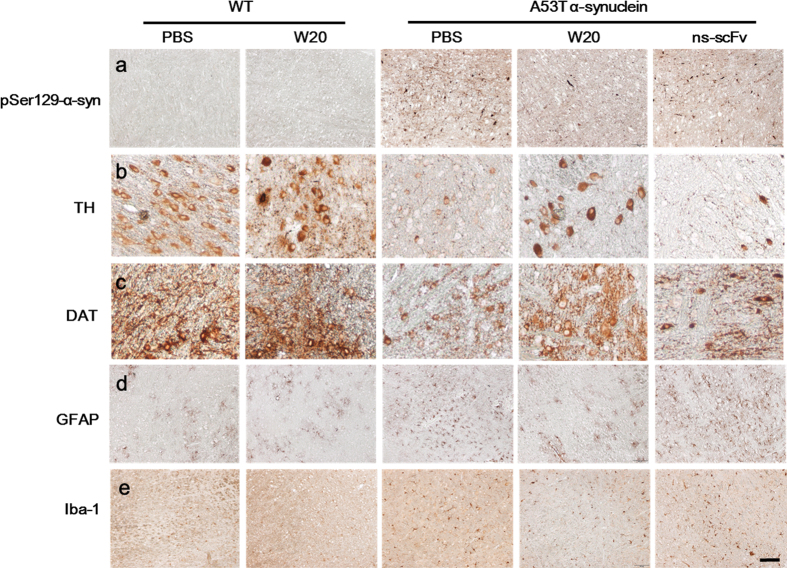
W20 attenuated neuropathology and gliosis in the brains of A53T α-synuclein mice. Phospho-Ser129-α-synuclein (**a**), tyrosine hydroxylase (TH, **b**), the dopamine transporter (DAT, **c**), GFAP (**d**) and Iba-1 (**e**) immunostaining in the brainstem of WT and A53T α-synuclein mice treated with or without W20 was detected. *n* = 6 mice/group. Scale bar, 100 μm.

To further explore the effects of W20 on the different neurodegenerative phenotype, we assessed the extent of neuropathology of A53T α-synuclein mice by immunohistochemistry analysis. Human α-synuclein levels in the brainstem of A53T α-synuclein mice were further detected by immunostaining with an antibody to detect phospho-Ser129-α-synuclein, which is a specific pathological form of human α-synuclein. The results showed that phospho-Ser129-α-synuclein positive staining was clearly detected throughout the brainstem of A53T α-synuclein mice rather than WT mice. By contrast, W20, but not ns-scFv, significantly reduced α-synuclein levels (Fig. 4a, Supplementary Fig. 2a).

Levels of tyrosine hydroxylase (TH) and dopamine transporter (DAT) were quantified to evaluate the effect of W20 on the dopaminergic (DA) signaling pathway. TH is the limiting enzyme in DA synthesis^32^, whereas DAT is a plasma membrane glycoprotein expressed in DA cells, responsible for DA uptake through a Na^+^/Cl^−^ coupled cotransport mechanism^33^. Both of them are pivotal players in DA signaling. A significant negative correlation was found between TH and DAT expression and motor impairment^31^,^32^. TH and DAT levels were significantly decreased in the brainstem of A53T α-synuclein mice compared with those of the WT littermates, whereas W20, not PBS or ns-scFv, remarkably increased TH and DAT levels in PD transgenic mice (Fig. 4b,c, and Supplementary Fig. 2b,c). These results indicated that W20 substantially attenuated neuropathology in A53T α-synuclein mice.

### W20 attenuated gliosis in the brains of A53T α-synuclein mice

Increased inflammation in the brains of A53T α-synuclein mice is strongly correlated with motor and memory impairment^34^,^35^. We detected the effects of W20 on astrocytosis and microgliosis in the brainstem of A53T α-synuclein mice. The results showed that astrocytosis in PD transgenic mice significantly increased compared with that in WT mice, and W20, rather than ns-scFv, remarkably reduced astrocytosis (Fig. 4d, Supplementary Fig. 2d). Similarly, A53T α-synuclein mice exhibited increased activation of microglia, whereas W20, but not ns-scFv, markedly decreased microgliosis (Fig. 4e, Supplementary Fig. 2e).

### W20 ameliorated motor and memory impairments in BACHD mice

**Figure 5:**
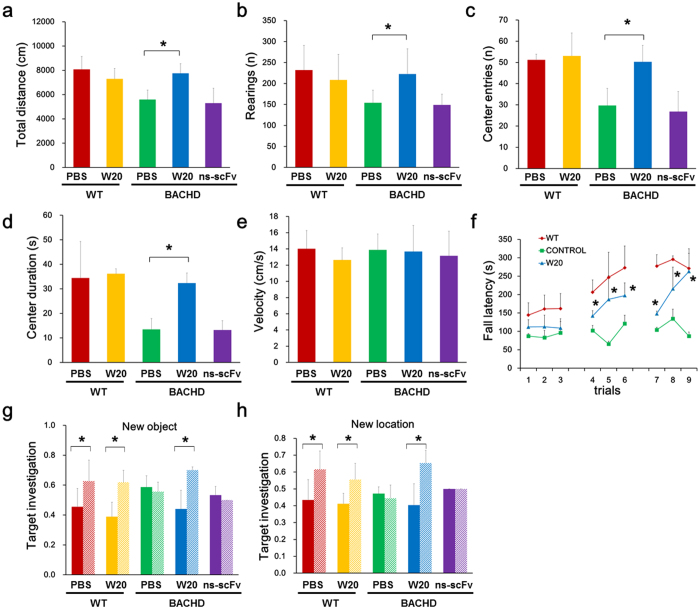
W20 ameliorated motor and memory impairments in BACHD mice. (**a–e**) The locomotion and anxiety behavior of WT and BACHD mice was assessed by open field test. Total distance traveled (**a**), rearing frequency (**b**), center entries (**c**), total time spent in the center of the field (**d**), and mean velocity during the exploration period (**e**) were measured. *n* = 8 mice/group. *P < 0.05, one-way ANOVA followed by Tukey’s *post hoc* test. (**f**) Rotarod tests were conducted in nine accelerating rotarod trials over 3 consecutive days. The average latency to fall was determined. *n* = 8 mice/group. *P < 0.05, one-way ANOVA followed by Tukey’s *post hoc* test. The cognition of mice treated with or without W20 was measured by assessing the object recognition using the novel object preference (**g**) and novel object location assays (**h**). *n* = 8 mice/group. *P < 0.05, one-way ANOVA followed by Tukey’s *post hoc* test. Data represent means ± SD.

BACHD mouse is a conditional human genomic transgenic mouse model for HD, which expresses human full-length mHTT with a polyQ repeat of 97 glutamines^36^. Model mice exhibit progressive motor and psychiatric-like behavioral deficits, as well as selective cortical and striatal atrophy^37^, and are thus considered suitable for HD preclinical studies. The open field test is a reliable assay for evaluating locomotion and anxiety in BACHD mice. During the 30-minute open field exploration, vehicle-treated or ns-scFv-treated BACHD mice displayed hypoactivity and anxiety by covering a low total distance (Fig. 5a), showing limited rearing (Fig. 5b), entering the center of the field infrequently (Fig. 5c), and spending a limited amount of time at the center of the field (Fig. 5d). By contrast, W20 treatment significantly decreased hypoactivity and anxiety-like behavior by increased locomotion (Fig. 5a) and rearing (Fig. 5b), higher frequency of entering the center (Fig. 5c) and more time spent in the center (Fig. 5d). In addition, all mice, regardless of genotype or treatment, traveled at similar average velocities (Fig. 5e). These results demonstrated that W20 could markedly improve locomotion and attenuate exploration deficits in BACHD mice.

Progressive motor deficit is an important clinical feature of HD^36^. The accelerating rotarod test was performed to evaluate the therapeutic efficacy of W20 on motor performance in BACHD mice. At 12 months of age, BACHD mice showed robust rotarod impairment compared with the WT littermates in all trials. However, W20-treated BACHD mice stayed on the accelerating rotarod longer during each individual testing session than vehicle-treated BACHD mice (Fig. 5f). These findings indicated that W20 rescued motor impairment in BACHD mice.

Cognitive impairment, in addition to motor abnormalities, is another important clinical characteristic of HD patients^38^. We performed the object recognition test to verify whether W20 treatment could improve the cognitive function in BACHD mice. BACHD mice treated with vehicle or ns-scFv exhibited cognitive deficits in novel object recollection, whereas W20 treatment significantly prevented the cognitive impairment in BACHD mice (Fig. 5g,h).

### W20 reduced mHTT levels in BACHD mouse brains

**Figure 6:**
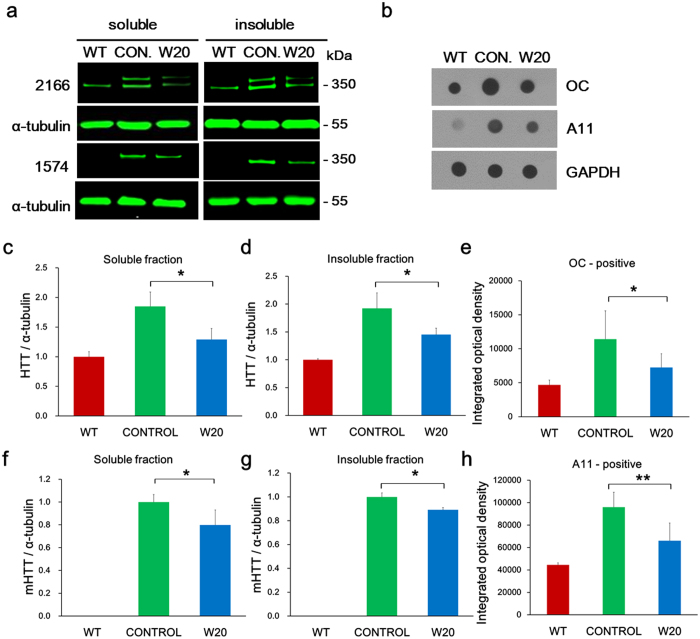
W20 reduced mHTT levels in BACHD mouse brains. (**a**) Western blot was performed to detect both total HTT and mHTT levels in soluble and insoluble fraction of WT and BACHD mouse brain lysates. HTT protein signal intensity for each sample was normalized to α-tubulin protein signal intensity. (**b**) OC-positive FOs and A11-positive PFOs levels in soluble fraction of WT and BACHD mouse brain lysates were detected by dot-blot. Oligomer signal intensity for each sample was normalized to GAPDH signal intensity. Total HTT (**c,d**) and mHTT levels (**f,g**) in soluble (**c,f**) and insoluble fraction (**d,g**) of WT and BACHD mouse brain lysates were quantitatively analyzed. OC-positive FOs (**e**) and A11-positive PFOs (**h**) levels in soluble fraction of WT and BACHD mouse brain lysates were quantitatively analyzed. *n* = 6 mice/group. *P < 0.05, **P < 0.01, Student’s *t*-test. Data represent means ± SD.

To further address the mechanism by which W20 exerted protective effect, we performed western blot to evaluate the relative levels of mHTT in both RIPA-soluble fraction and 10% SDS-soluble fraction, the latter contained the mHTT aggregates from the mouse brain lysates and were termed as insoluble fraction. Levels of soluble and insoluble mHTT were significantly higher in the BACHD mouse control than in the WT littermates (Fig. 6a,c,d,f,g). Treating BACHD mice with W20 markedly decreased both soluble and insoluble mHTT levels in the BACHD mice brains. While neither ns-scFv affected the mHTT levels in the brains of BACHD mice nor W20 had any effects in WT mice, suggesting W20 had specific therapeutic function on HD mice (Supplementary Fig. 3).

We also determined the levels of mHTT oligomers in the mouse brain lysates by dot-blot using oligomer-specific antibodies OC and A11 (Fig. 6b). W20-treated mice showed 36.5% (Fig. 6e) and 31.1% (Fig. 6h) reductions in the levels of mHTT oligomers, as detected by OC and A11 antibodies, respectively, compared with vehicle-treated BACHD mice.

### W20 reduced neuropathology in BACHD mice

**Figure 7:**
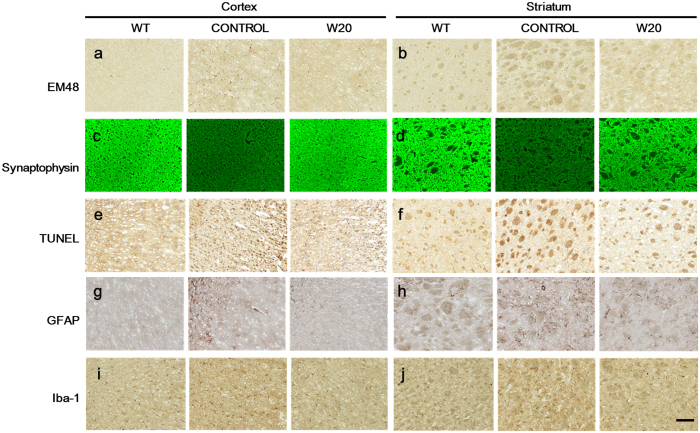
W20 reduced neuropathology and neuroinflammation in BACHD mice. EM48 (**a,b**) and synaptophysin (**c,d**) immunostaining, TUNEL analysis (**e,f**), GFAP (**g,h**) and Iba-1 (**i,j**) immunostaining in the cortex and striatum of WT and BACHD mice treated with or without W20 were performed. *n* = 6 mice/group. Scale bar, 100 μm.

Progressive aggregation of mHTT in the brain is another pathological hallmark of HD. The BACHD mouse model may reproduce a mHTT aggregation pattern similar to that in adult-onset HD and exhibits robust high levels of mHTT aggregates at 12 months of age^36^. Here we examined the effects of W20 on mHTT aggregates in the cortex and striatum of BACHD mice by EM48 immunostaining. The results showed that mHTT aggregates dramatically decreased in the cortex and striatum of W20-treated BACHD mice compared with those in the vehicle-treated BACHD control by 26% (Fig. 7a, Supplementary Fig. 4a) and 49.5% (Fig. 7b, Supplementary Fig. 4b), respectively.

Levels of synaptophysin, a presynaptic marker, in mouse brains were measured by immunostaining to further explore the mechanism by which W20 attenuated motor and memory deficits in BACHD mice. Vehicle- or ns-scFv-treated BACHD mice exhibited obvious loss of synaptophysin in the cortex and striatum, whereas W20 significantly increased synaptophysin levels in BACHD mice, but not WT mice (Fig. 7c,d, Supplementary Figs 4c,d and 5), thereby indicating that W20 treatment may rescue neurodegeneration by inhibiting synapse loss in the brains of BACHD mice.

Neuron death is an important pathological hallmark in HD. We performed TUNEL analysis on the brain sections of BACHD mice to assess the neuronal protective effect of W20. The number of apoptotic cells in the brains of vehicle-treated BACHD mice was significantly higher than that in the WT littermates (Fig. 7e,f). W20 treatment decreased the apoptotic cells by 69.6% (Supplementary Fig. 4e) in the cortex and 73.3% (Supplementary Fig. 4f) in the striatum of BACHD mice.

### W20 attenuated the neuroinflammation in BACHD mice

Increasing evidence reveals that reactive gliosis occurs in vulnerable regions of HD brains and strongly associated with HD pathogenesis^39^. In this study, we evaluated the effects of W20 on astrocytosis and microgliosis in BACHD mouse brains by immunostaining for GFAP and Iba-1. The brains of BACHD mice showed substantial astrocytosis and microgliosis at 12 months of age, whereas W20 significantly inhibited this neuroinflammation (Fig. 7g–j). Immunostaining area analysis demonstrated that W20 decreased the astrocytosis in the cortex and striatum by 55.7% (Supplementary Fig. 4g) and 72.7% (Supplementary Fig. 4h), respectively, and reduced microgliosis by 15.2% (Supplementary Fig. 4i) and 28.1% (Supplementary Fig. 4j), respectively. Additionally, ns-scFv, unlike W20, did not affect the astrocytosis and microgliosis in the BACHD mice, W20 also did not affect the levels of GFAP and Iba-1 in WT mice (Supplementary Fig. 5), confirming the therapeutic specificity of W20 on HD mice.

### W20 decreased TNF-α and IL-1β production in BACHD mice

Proinflammatory cytokines contribute to the neurodegeneration in HD patients and mouse models^40^. To investigate the effect of W20 on cytokine production, we determined TNF-α and IL-1β levels in the brain lysates of BACHD mice treated with or without W20. TNF-α and IL-1β levels were significantly reduced in W20-treated BACHD mice compared with those in vehicle-treated mouse controls by 13.1% (Supplementary Fig. 6a) and 16.8% (Supplementary Fig. 6b), respectively. These results suggest that W20 may exert anti-neuroinflammatory effect by downregulating cytokine production.

### W20 prevented ROS generation and increased SOD activity in the brains of BACHD mice

Oxidative stress is a fundamental aspect of HD pathogenesis^41^. We determined ROS levels in the brain lysates of BACHD mice to evaluate the effects of W20 on ROS production. ROS levels were markedly increased in the brains of vehicle-treated BACHD mice compared with those in the WT littermate controls. However, W20 treatment prevented this increase by 27.4% (Supplementary Fig. 6c). We then determined the SOD activities in the brain lysates to examine whether W20 affected the antioxidant activities in the brains of BACHD mice. A significant increase in total SOD (Supplementary Fig. 6d) and Mn-SOD (Supplementary Fig. 6e) activity was observed in W20-treated BACHD mice relative to the vehicle-treated BACHD controls.

### W20 penetrated neurons and dissociated performed amyloid deposits

We previously reported that W20 disrupted the equilibrium between oligomers and fibrils, and disassociated preformed amyloid fibrils *in vitro*^26^. To validate whether W20 penetrated the neurons, mouse hippocampal neuronal cells (HT22) were incubated with W20 for 2 h, and then the distribution patterns of W20 were visualized by confocal laser-scanning microscopy. The results showed that W20 was internalized and located in the cytoplasm, but not in the nuclear region of HT22 cells (Supplementary Fig. 7). To investigate the potential mechanism for W20 to reduce intracellular amyloid deposits, the brain slides of A53T α-synuclein mice and BACHD mice were incubated with W20 (2 mg/ml) overnight at 37 °C, and then the aggregates of human α-synuclein and mHTT were detected by phospho-Ser129-α-synuclein and EM48 immunostaining, respectively. The results showed that both α-synuclein and mHTT aggregates were significantly decreased in the brain slides, suggesting W20 may decrease intracellular amyloid deposits in transgenic mouse models by penetrating neurons and disassociating preformed amyloid aggregates (Supplementary Fig. 8).

### Binding of W20 to α-synuclein

**Figure 8:**
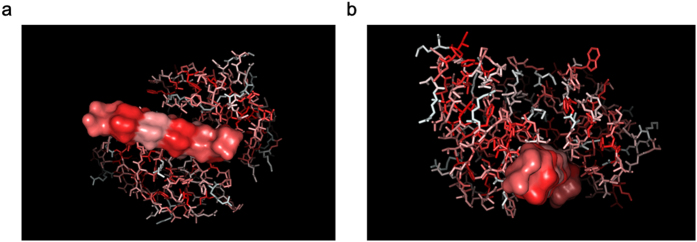
Binding of W20 to α-synuclein dimer by molecular dynamics simulation. ScFv antibody structure was modeled by customized program through homology modeling, protein-peptide docking was performed using AutoDock. One ns molecular dynamics simulation was carried out at a time step of 2.0 fs. W20 predominately binds to Val 71 and Val 77 of α-synuclein dimer through hydrophobic interaction with the residues in CDR3 region of W20 (hydrophobic amino acids are indicated in red). (**a**) Top-side view, (**b**) rear-side view.

Previous report indicated that oligomer-specific scFv antibody W20 recognized α-synuclein oligomers^26^. To determine which residues in α-synuclein dimer contribute to the binding to W20, we performed molecular dynamics analysis. The results showed that W20 predominately binds to Val 71 and Val 77 of α-synuclein dimer through hydrophobic interaction with the residues in its CDR3 region (Fig. 8a,b).

## Discussion

Passive immunization has emerged as the main immunotherapy strategy against neurodegenerative diseases because of the side effects and poor responses of elderly patients to vaccination^42^. To the best of our knowledge, our study is the first to evaluate the implications of passive immunotherapy with oligomer-specific antibody in both PD and HD transgenic mouse models simultaneously.

Amyloid aggregation is a molecular pathogenic mechanism for numerous age-dependent neurodegenerative disorders, such as AD, PD, and HD. Although the symptoms, pathology, and related amyloids of different amyloidoses are very diverse, amyloid aggregates share common features such as cross-β-sheet structures, common epitopes, and neurotoxicity. As well, amyloid aggregates have oligomer-specific or fibril-specific epitopes^8^. Amyloid oligomers play a key role in the pathological processes of amyloidoses^7^. Oligomers formed from α-synuclein, mHTT, and Aβ may induce oxidative stress, gliosis, synapse loss, and neuron death^9^. Targeting these oligomers could be a direct and efficient approach for treating amyloidoses. Different amyloids, such as Aβ, PrP, α-synuclein, polyQ, and amylin, not only aggregate into oligomers with common epitopes, but also promote the aggregation of other amyloids, mutually enhance the cytotoxicity of amyloid aggregates, and facilitate the progression of amyloidoses^13^,^14^,^43^. Thus, strategies targeting common oligomeric conformation would be an appealing approach to circumvent oligomer-mediated cytotoxicity by various amyloids, such strategies are widely believed to be a more efficient treatment method for amyloidoses than agents targeting a single type of amyloid. Moreover, the approach may have a broader spectrum to treat different amyloidoses regardless of the different sequences of disease-associated amyloids. We previously reported an oligomer-specific scFv antibody, W20, recognized the common epitope of oligomers assembled from Aβ, α-synuclein, insulin, lysozyme, and amylin, and inhibited their cytotoxicity^26^. When applied to the AD transgenic animal model, W20 prevented memory deficits and attenuated pathologies in APP/PS1 transgenic mice^28^. In the present study, we tested the effects of W20 on PD and HD transgenic mouse models, and our results indicated that W20 ameliorated motor deficits in PD and HD transgenic mouse models by improving motor cooperation. Moreover, the spatial memory deficits of PD and HD transgenic mice were significantly improved, as detected by the object recognition test and MWM test.

Soluble oligomer species are typically transient and structurally heterogeneous. Several types of α-synuclein oligomers are found in the brains of PD patients and transgenic mice, which may be classified as OC-positive fibrillar oligomers (FOs) or A11-positive prefibrillar oligomers (PFOs)^12^. W20 not only significantly reduced the levels of both types of α-synuclein oligomers, but also decreased α-synuclein deposits in the brainstem of A53T α-synuclein mice. This finding is consistent with a previous report that W20 disrupts the equilibrium between oligomers and fibrils, and disassociates preformed fibrils^26^. Similarly, our findings showed that W20 treatment significantly reduced levels of soluble and insoluble mHTT, mHTT oligomers as well as EM48-positive mHTT aggregate accumulation in the brains of BACHD mice. Numerous processes, such as aberrant cleavage of amyloid proteins, protein misfolding, and reduced protein degradation because of disruption of autophagy and/or the ubiquitin-proteasome system, contribute to amyloid aggregate formation^44^, W20 may block α-synuclein and mHTT fibrillation, reduce their cytotoxicity, and accelerate amyloid oligomer clearance from transgenic mouse brains, resulting in improved motor and cognition performances.

α-Synuclein plays a role in synaptic transmission, axonal transport, and regulation of DA homeostasis^45^. TH is a rate-limiting enzyme involved in DA generation, while DAT is expressed in striatal presynaptic terminals and is a major determinant of DA homeostasis. TH and DAT levels are inversely correlated with the total α-synuclein burden in the substantia nigra of PD patients or animal models^32^,^46^. W20 increased levels of TH and DAT in the brainstem of A53T α-synuclein mice by decreasing α-synuclein levels, protecting neuronal cells from degeneration, thus leading the improvement of motor and cognition performances. Synaptic deficiencies likely contribute to the clinical symptoms in HD patients, such as chorea, dystonia, and cognitive decline^23^. Moreover, mHTT oligomers play a key role in causing neuronal cell death in HD by activating apoptotic pathways^47^. W20 decreased neuronal apoptosis, resulting in increased synaptophysin levels in the cortex and striatum of BACHD mice.

α-Synuclein and mHTT aggregates induced neuroinflammatory reactions, especially astrocytosis and microgliosis in the brains of PD and HD patients and corresponding mouse models, which contribute significantly to the progression of PD and HD^48^,^49^. We observed a positive correlation between levels of α-synuclein or mHTT accumulation and levels of GFAP and Iba-1 in the brains of A53T α-synuclein and BACHD mice, indicating that pathogenic amyloid aggregates could be correlated with the degree of neuroinflammation. W20 treatment markedly attenuated astrocytic and microglial activation by decreasing levels of α-synuclein and mHTT aggregates. As immunotherapy for CNS disorders is likely to induce severe adverse neuroinflammatory responses^50^, which is important for the development of neurodegenerative diseases, thus, an immuno-agent for treating CNS disorders should not promote neuroinflammation. In this study, W20 exhibited its beneficial neuro-protective effects by alleviating astrocytosis and microgliosis.

Proinflammatory cytokines, such as TNF-α and IL-1β, are elevated in HD brains and may augment inflammatory signals^51^, contributing directly to neuronal dysfunction. Treating BACHD mice with W20 significantly reduced the production of proinflammatory factors by neutralizing toxic mHTT oligomers. In addition, oxidative stress in cells occurs under excess ROS levels or deficient antioxidant capacity, causing morphological abnormalities and death in neurons^52^. The brains of HD patients and mouse models have been reported to show signs of oxidative stress and DNA damage^41^,^53^. W20 markedly decreased ROS production and normalized SOD activity, thus suggesting that W20 could protect the brains of BACHD mice from oxidative stress through blocking mHTT oligomer-induced neuronal cytotoxicity.

In conclusion, a therapeutic approach using W20 against a common epitope of various toxic oligomeric structures assembled from different amyloids significantly improved motor and cognitive deficits in PD and HD transgenic mice. Moreover, levels of pathogenic α-synuclein and mHTT species were reduced and neuroinflammation in the mouse brains was attenuated as well. In our recent study, 3F, the matured form of W20, exhibited remarkably increased affinity to pathogens, longer half-life period *in vivo*, better penetration across the blood brain barrier, and beneficial effects on cognitive improvement in AD mice by intranasal delivery, further showing the promising potential of a disease mechanism-directed clinical therapy against PD, HD, AD, and other amyloidoses.

## Methods

### Animals and W20 treatment

9-month-old male A53T α-synuclein mice, 12-month-old male BACHD mice, and their WT littermates were purchased from Jackson Laboratory (Stock numbers are 006823 and 008197, respectively). A53T α-synuclein mice overexpress human A53T α-synuclein using the mouse prion protein promoter. BACHD mice are bacterial artificial chromosome-mediated transgenic models that express full-length human mHTT with 97 CAG repeats^36^. All mice for experiments were group-housed (five mice/cage), provided food and water ad libitum, and kept in a colony room at 22 ± 2 °C and 45% ± 10% humidity on a reverse 12 h light/dark cycle. W20 was developed and prepared in our laboratory as previously described^27^. An ns-scFv antibody was obtained from a phage library and showed no affinity to α-synuclein or mHTT. All of the mice were anesthetized intraperitoneally with avertin (300 mg/kg) and placed in a stereotaxic apparatus. Intracerebroventricular (icv) administration was conducted according to a previously described method with minor modifications^54^. An incision was made in the scalp of each mice. The skull was exposed, and burr holes were drilled in the skull over the injection site. The injection coordinates were 1.8 mm caudal to bregma, 1.8 mm lateral to midline, and 2.5 mm ventral to the brain surface of the skull. A Hamilton microsyringe fitted with a 30-gauge needle was used for icv infusion of W20 (5 μL, 4 mg/mL), ns-scFv (5 μL, 4 mg/mL), or PBS (5 μL) as the vehicle control, injections were administered at a rate of 0.2 μL/min. After injection, the needle was retained for 5 min to ensure adequate diffusion of agents and then was slowly retracted. Another icv injection was conducted after 7 d. Mice were randomly treated in cohorts (n = 8) to render behavior testing more manageable. No mice were excluded from behavioral studies due to fever, weight loss, infection or behavioral alterations as a result of W20 treatment. All experiments were performed in accordance with the China Public Health Service Guide for the Care and Use of Laboratory Animals. Experiments involving mice and protocols were approved by the Institutional Animal Care and Use Committee of Tsinghua University.

### Open field exploration

A53T α-synuclein and BACHD mice were placed in the center of the chamber (27 × 27 × 20.3 cm^3^), and their behavior was recorded for 15 and 30 min, respectively. Five measures, namely, total locomotion, total rearing frequency, center entries, center duration and velocity were quantitatively analyzed. Chambers were cleaned with 70% ethanol before each use.

### Rotarod test

Motor coordination was assessed with a rotarod apparatus (TSE Systems). Daily sessions included a 5-minute training trial at 4 rpm. After 1 h, the animals were tested over three consecutive accelerating trials with the speed changing from 0 rpm to 40 rpm over 300 s. The inter-trial interval was 30 min. The latency to fall from the rod was recorded, and results were averaged. Mice remaining on the rod for more than 300 s were removed, and their time was scored as 300 s. Mice were tested over three consecutive days.

### Hindlimb clasping

Hindlimb clasping was evaluated in A53T α-synuclein mice according to a previously described method^55^. Mice were suspended by the base of the tail and videotaped for 15 s. Hindlimb clasping was rated from 0 to 3 based on severity: 0 = hindlimbs splayed outward and away from the abdomen; 1 = one hindlimb retracted inward toward the abdomen for at least 50% of the observation period; 2 = both hindlimbs partially retracted inward toward the abdomen for at least 50% of the observation period; and 3 = both hindlimbs completely retracted inward toward the abdomen for at least 50% of the observation period. Scores of 0.5 were utilized when appropriate. Hindlimb clasping severity scores were averaged for the three separate trials over three consecutive days.

### Pole test

The pole test was utilized to measure motor coordination and balance in A53T α-synuclein mouse model. Mice were initially habituated and trained 1 d prior to testing. Then they were placed on the top of a rough-surfaced wooden pole (50 cm in length and 1 cm in diameter) and allowed to descend to the base of the pole. During the test, mice were placed with their heads oriented toward the top of the pole. The time required by the mouse to turn its head downward and descend the entire length of the pole was measured. The best performance of each mouse over five consecutive trials was recorded.

### MWM test

The effect of W20 on the spatial cognitive performance of transgenic mice were investigated through the MWM test according to a previously described method^56^. The mice were allowed to habituate for 1 day and then tested in a water maze (1.1 m in diameter). The maze filled with water was drained daily. The temperature of the water was maintained at 22 ± 1 °C. The platform (10 cm in diameter) was fixed 1 cm beneath the water surface throughout the training period, and the starting positions were counter balanced. All mice were initially assessed in the water maze to identify inherent quadrant preferences, and mice exhibiting some preferences were eliminated from subsequent testing. The mice were allowed to swim for 60 s to find the platform, on which they were allowed to stay for 10 s. Mice unable to locate the platform were guided to it. The mice were trained twice per day over five consecutive days, with an inter-trial interval of 3–4 h. The swimming activity of each mouse was monitored using a video camera (Sony, Tokyo, Japan) mounted over the maze and automatically recorded via a video tracking system. About 24 h after the last learning trial, the mice were tested for memory retention in a probe trial without the platform.

### Object recognition

The object recognition test was performed according to the previously described method with slight modifications^57^. In the novel location task, mice were first presented with two different novel objects placed in the upper two corners of a box (50 cm × 50 cm × 25 cm) for 5 min. Then, the mice were removed from the box for a 1 h retention period, and the upper right object was moved to the lower right corner. Afterward, the mice were reintroduced to the box, and their behavior was recorded for 5 min. The percentages of investigations to the target object in the new and original locations were recorded. Preference for a novel object was assessed on the subsequent day with the exception that the retention period was 24 h. The right object was replaced with a completely novel object in the original location.

### Brain lysate preparation and Western blot

The brain tissues of A53T α-synuclein mice were homogenized in TNE buffer (10 mM Tris-HCl, pH 7.4, 150 mM NaCl, 5 mM EDTA) containing complete protease inhibitor mixture tablets (Roche Diagnostics) and detergents (0.5% Nonidet P-40). The homogenate was centrifuged (5 min at 100,000× *g*), and the supernatants were collected for α-synuclein detection.

BACHD mouse brain lysates were prepared by homogenizing brain tissues in a modified RIPA buffer supplemented with complete protease inhibitor mixture tablets (Roche Diagnostics), then followed by centrifugation at 4 °C for 15 min at 16,000× *g*. The supernatants were obtained for soluble huntingtin protein (HTT) detection. The pellets were dissolved in 10% SDS, heated to 70 °C for 10 min, and then spun at 2000× *g* for 30 s. The new supernatants were considered to be the insoluble fraction.

The protein concentrations of soluble and insoluble fractions were determined using the BCA protein assay (Pierce) according to the manufacturer’s instructions. Proteins were separated by SDS-PAGE and transferred onto a nitrocellulose membrane (Bio-RAD). α-synuclein immunoblot analysis was performed using primary antibodies including Syn-1 (BD Biosciences, 610787, 1:1000) against both human and mouse α-synuclein, and LB509 (Abcam, ab27766, 1:1000) against human-specific α-synuclein. For HTT immunoblot analysis, the membranes were blotted with primary antibodies including 2166 (Merck Millipore, MAB2166, 1:1000) against both mHTT and endogenous WT-HTT, and 1574 (Merck Millipore, MAB1574, 1:1500) against polyQ-expanded HTT. α-tubulin (Sigma, T9026, 1:1000) was used as a loading control. Blots were washed thrice in TBST before incubation with IR secondary antibodies (Li-Cor, #926-3211 and #926-68020, 1:5000) for 1 h at room temperature. Then blots were washed thrice again and imaged in a Li-Cor Odyssey IR detection system. Densitometry was performed using the integrated intensity value for each band, and the results were analyzed as the ratio of protein to α-tubulin.

### Dot-blot

The soluble fraction of brain homogenates was applied to nitrocellulose membranes (Merck Millipore), which were then blocked with 5% milk in PBST for 1 h and incubated with OC (Merck Millipore, SPC-507D, 1:1000), A11 (Invitrogen, AHB0052, 1:1000), or anti-GAPDH antibodies (CST, 2118S, 1:1000) at room temperature for 1 h. Next, the bound antibodies were probed with the corresponding HRP-conjugated secondary antibody. Immuno-reactive blots were visualized with an ECL chemiluminescence kit.

### Immunohistochemistry

Mice were deeply anesthetized with avertin, transcardially perfused with ice-cold PBS containing heparin (10 U/mL), and finally sacrificed. Their brains were immediately removed and divided along the sagittal plane. One brain hemisphere was fixed in 4% paraformaldehyde in PBS at 4 °C overnight and processed for either frozen or paraffin-embedded sections. For A53T α-synuclein mice, 6 μm sagittal paraffin-embedded sections were immunostained with anti-TH antibody (Abcam, ab152, 1:100), anti-DAT antibody (Merck Millipore, MAB369, 1:100), anti-phospho-Ser129-α-synuclein antibody (Abcam, ab59264, 1:50), anti-Iba-1 antibody (GeneTex, GTX100042, 1:100), or anti-GFAP antibody (Abcam, ab53554, 1:100), respectively. For BACHD mice, 20 μm coronal frozen sections were immunostained with anti-EM48 antibody (Merck Millipore, MAB5374, 1:50), anti-Iba-1 antibody (GeneTex, GTX100042, 1:100) or anti-GFAP antibody (Abcam, ab53554, 1:100), respectively. All aforementioned primary antibodies were followed by appropriate HRP-labeled secondary antibodies and visualized with diaminobenzidine (DAB). For synaptophysin staining, sections were immunostained with anti-synaptophysin antibody (Abcam, ab32127, 1:1000) followed by goat anti-rabbit secondary antibody conjugated to Alexa Fluor 488 (Santa Cruz, I1112, 1:1000).

All images were acquired with an Olympus IX73 inverted microscope with DP80 camera, and all analyses were performed blind to the genotype and treatment of the mice. We selected sections with similar neuroanatomical regions of the brainstem for A53T α-synuclein mice and similar neuroanatomical regions of the cortex or striatum for BACHD mice for all histological analyses. At least three sections were analyzed per mouse, and three fields of view for each section were imaged. Immunofluorescence intensities or immunostaining regions were quantified using Image J software (National Institutes of Health, USA).

For quantitative analysis of astrocytosis and microgliosis, we quantified the positively stained area in brainstem sections of PD mouse model, and cortex and striatum sections of HD mouse model. At least three sections were analyzed per mouse, and three fields for each section were imaged and quantified. GFAP or Iba-1 immunostaining regions were quantified using Image J software.

### TUNEL assay

Cell death was detected with an *in situ* cell death detection kit (Roche Diagnostics) according to the manufacturer’s protocol. Briefly, the brain sections were fixed, permeabilized, and incubated with 50 μLof TUNEL reaction mixture for 60 min at 37 °C in the dark. The sections were then rinsed by PBS thrice and incubated with 50 μL of converter-POD for 30 min. Sections were stained with DAB, counterstained with hematoxylin, and then imaged using an Olympus IX73 inverted microscope. Images were analyzed by Image J software.

### Molecular dynamics simulation

Molecular dynamics stimulation was calculated as previously described^58^,^59^. Initially, an unfolded and fully extended structure was generated by PROTEIN program in Tinker software^60^ (backbone torsion ϕ, ψ = −135, 135) using OPLS-AA force field parameters to assign the atom types. The structure was subsequently optimized by Truncated Newton Conjugate Gradient method using GB/SA continuum solvation model. Afterward, 1 ns molecular dynamics stimulation was carried out at a time step of 2.0 fs, and the system thermostat temperature was targeted to 298 K. The result of molecular dynamics stimulation was further rendered with POV-RAY program. ScFv antibody structure was modeled by customized program through homology modeling and protein-peptide docking was performed using AutoDock.

### Statistical analysis

All quantitative analysis was performed under blinded conditions. Statistical significance was tested using Student’s *t*-test or one-way ANOVA analysis of variance followed by Tukey’s *post hoc* test (GraphPad Prism 6.0). Results are expressed as group mean ± SD, and *P* < 0.05 was considered statistically significant.

## Additional Information

**How to cite this article**: Zha, J. *et al*. A scFv antibody targeting common oligomeric epitope has potential for treating several amyloidoses. *Sci. Rep.* **6**, 36631; doi: 10.1038/srep36631 (2016).

**Publisher’s note:** Springer Nature remains neutral with regard to jurisdictional claims in published maps and institutional affiliations.

## Supplementary Material

Supplementary Information
