# Supplementary material for: A scFv antibody targeting common oligomeric epitope has potential for treating several amyloidoses
Source: Sci Rep. 2016 Nov 8;6:36631. doi: 10.1038/srep36631 (PMC5100551; doi:10.1038/srep36631)
Supplement: Supplementary Information [file srep36631-s1.pdf]

A scFv antibody targeting common oligomeric epitope has potential for treating several amyloidoses

Jun Zha[1,2#], Xiang-meng Liu[1,3#], Jie Zhu[1#], Shu-ying Liu[1,4], Shuai Lu[1], Peng-xin Xu[4], Xiao-lin Yu[1,*], Rui-tian Liu[1,*]

[1] State Key Laboratory of Biochemical Engineering, Institute of Process Engineering, Chinese Academy of Sciences, Beijing, China

[2] School of Pharmacy, China Pharmaceutical University, Nanjing, China

[3] School of Bioengineering, Qilu University of Technology, Jinan, China

[4] School of Life Science, Ningxia University, Yinchuan, China

[#] These authors contributed equally to this work.

Corresponding to:

Rui-tian Liu,

State Key Laboratory of Biochemical Engineering,

Institute of Process Engineering, Chinese Academy of Sciences,

Haidian District, Beijing 100190, China

E-mail: rtliu@ipe.ac.cn, Tel.: +86 10 82545017; Fax: +86 10 82545025

Or: Xiao-lin Yu,

State Key Laboratory of Biochemical Engineering,

Institute of Process Engineering, Chinese Academy of Sciences,

Haidian District, Beijing 100190, China

E-mail: yuxiaolin@ipe.ac.cn, Tel.: +86 10 82545075; Fax: +86 10 82545025

Supplementary Method

Measurement of IL-1β and TNF-α. The IL-1β and TNF-α levels in the brain lysates were determined using IL-1β and TNF-α ELISA kits according to the manufacturer's protocols. Briefly, the brain lysates were added to a 96-well ELISA plate and then reacted with the relevant primary antibodies and HRP-conjugated secondary antibodies. 3,3,5,5-Tetramethylbenzidine was used as the substrate, and the absorbance of the mixtures was measured at 450 nm using an MD-M5 microplate reader.

Measurement of ROS and SOD. ROS assay was performed as previously described[1]. Briefly, ROS production was fluorimetrically monitored using 2',7'-dichlorofluorescein diacetate (DCFH-DA) mixed with the brain lysates of mice. The intensity of DCF fluorescence generated from carboxy-DCFDA was proportional to the amount of ROS. ROS units were determined using an MD-M5 microplate reader (excitation, 485 nm; emission, 530 nm).

The activity of SOD in the brain lysates was detected by a SOD assay kit according to the manufacturer's protocol[2]. Briefly, brain lysates, nitroblue tetrazolium, and enzyme-working solutions were prepared and added to a 96-well plate. The mixtures were incubated at 37 °C for 20 min, and their absorbance was assayed at 560 nm using an MD-M5 microplate reader.

Immunofluorescence. HT22 cells were exposed to W20 for 2 h, and then rinsed three times in PBS, fixed in 3.7% paraformaldehyde for 15 min at RT, permeabilized with 0.3% Triton X-100 in PBS for 15 min, and blocked for 1 h in 5% normal goat serum blocking solution. Cells were processed simultaneously for immunofluorescence using

9E10 antibody followed by the secondary antibody Alexa 488-conjugated anti-mouse IgG (1:1,000, Abcam). Nuclear DNA was counterstained with DAPI. Fluorescence signals were captured on a Carl Zeiss LSM780 laser scanning confocal microscope with a ×63 oil immersion (1.4 NA) objective. Images were processed using ZEN image software.

References

1. Zhou WW*, et al.* Decreasing oxidative stress and neuroinflammation with a multifunctional peptide rescues memory deficits in mice with Alzheimer disease. *Free Radic Biol Med* **74**, 50-63 (2014).

2. Xu PX*, et al.* Rutin improves spatial memory in Alzheimer's disease transgenic mice by reducing Abeta oligomer level and attenuating oxidative stress and neuroinflammation. *Behavioural brain research* **264**, 173-180 (2014).

Supplementary Figures

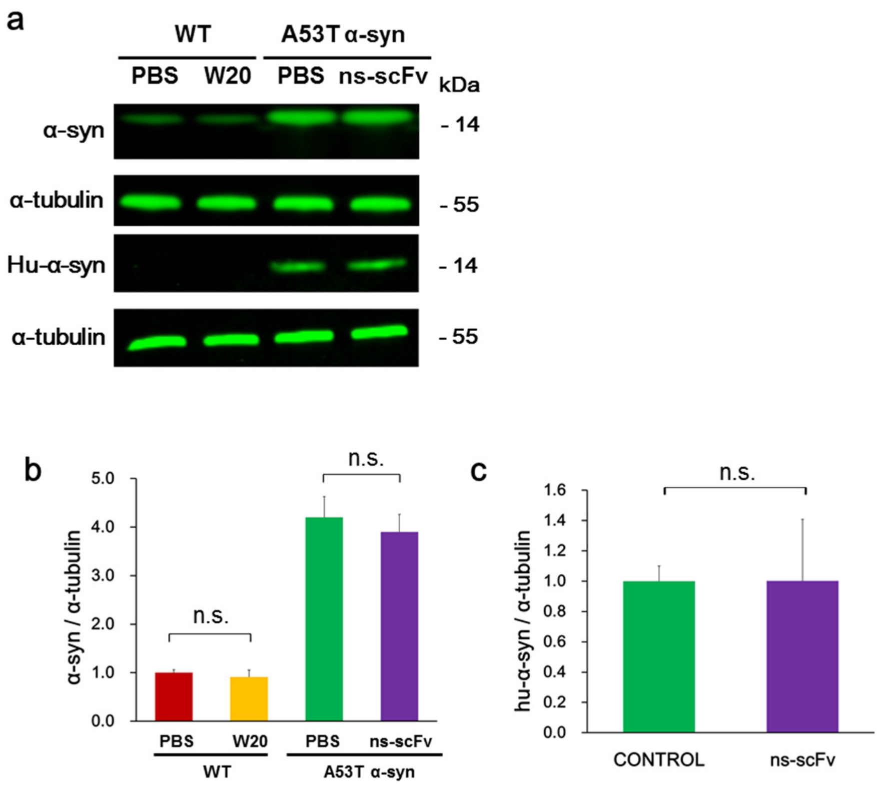

Supplementary Figure 1. Non-specific scFv did not affect α-synuclein levels in A53T α-synuclein mouse brains. (**a**) Western blot was performed to measure the levels of total α-synuclein and overexpressed human A53T α-synuclein levels in WT and A53T α-synuclein mouse brain lysates, respectively. Alpha-synuclein protein signal intensity for each sample was normalized to α-tubulin protein signal intensity. The levels of total α-synuclein (**b**) and overexpressed human A53T α-synuclein levels (**c**) in WT and A53T α-synuclein mouse brain lysates were quantitatively analyzed. n = 6 mice/group. n.s., not significant, Student's t-test. Data represent means ± SD.

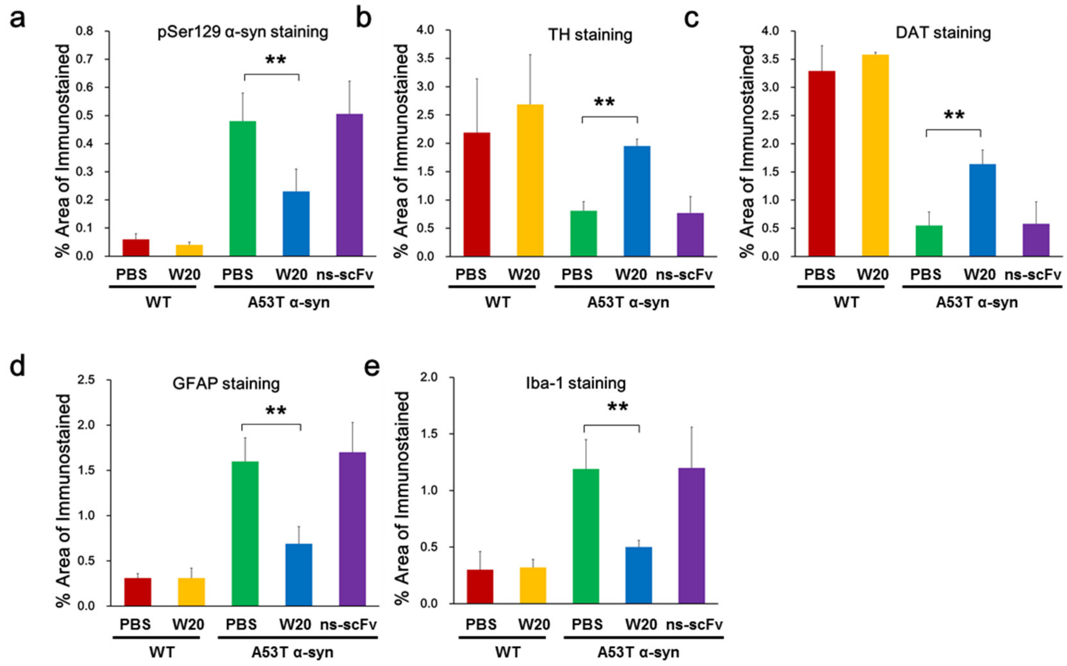

Supplementary Figure 2. W20 attenuated neuropathology and gliosis in the brains of A53T α-synuclein mice. Phospho-Ser129-α-synuclein (**a**), tyrosine hydroxylase (TH, **b**), the dopamine transporter (DAT, **c**), GFAP (**d**) and Iba-1 (**e**) immunostaining in the brainstem of WT and A53T α-synuclein mice treated with or without W20 were quantitative analyzed. n = 6 mice/group. **$P < 0.01$, Student's t-test. Data represent means ± SD.

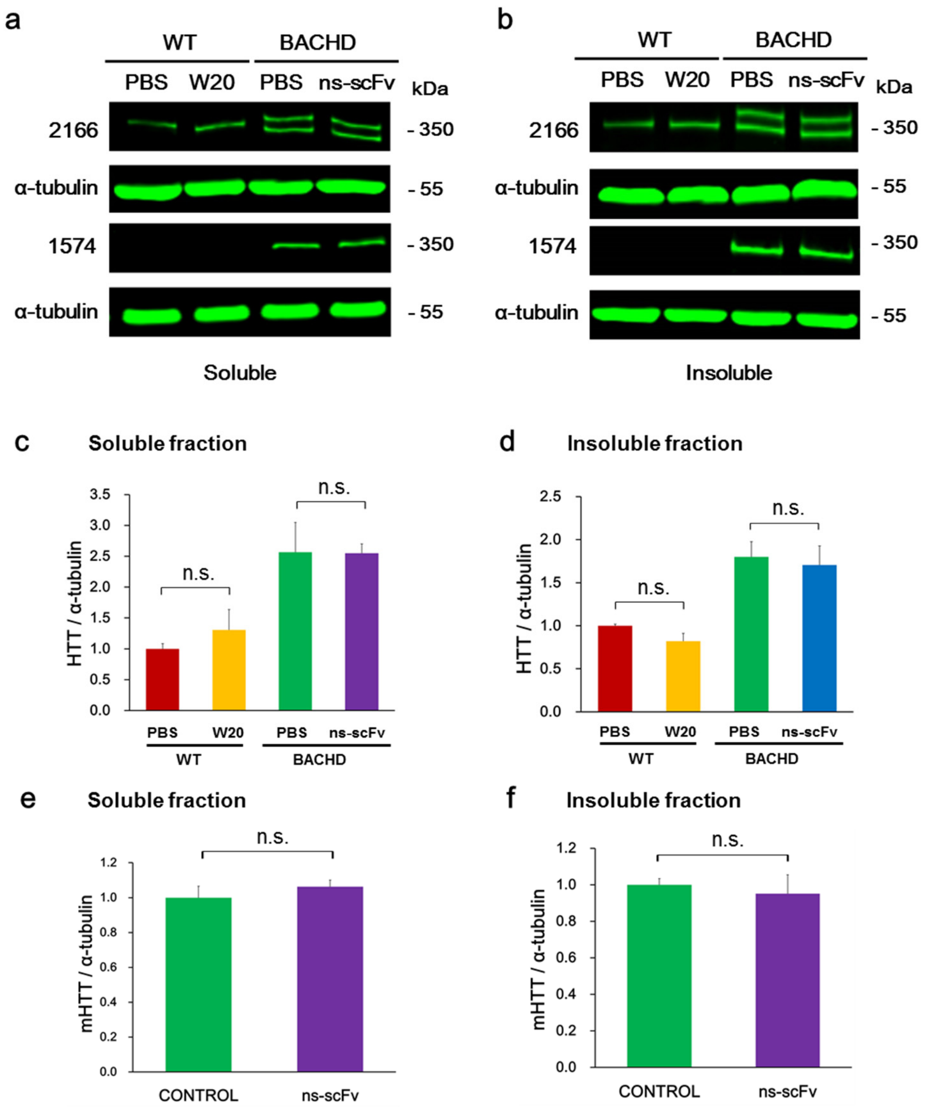

Supplementary Figure 3. Non-specific scFv did not affect HTT levels in BACHD mouse brains. (**a**) Western blot was performed to detect both total HTT and mHTT levels in soluble and insoluble fractions of WT and BACHD mouse brain lysates, respectively. HTT protein signal intensity for each sample was normalized to α-tubulin protein signal intensity. The levels of total HTT (**c, d**) and mHTT levels (**e, f**) in soluble (**c, e**) and insoluble fraction (**d, f**) of WT and BACHD mouse brain lysates were quantitatively analyzed. *n* = 6 mice/group. n.s., not significant, Student's t-test. Data represent means ± SD.

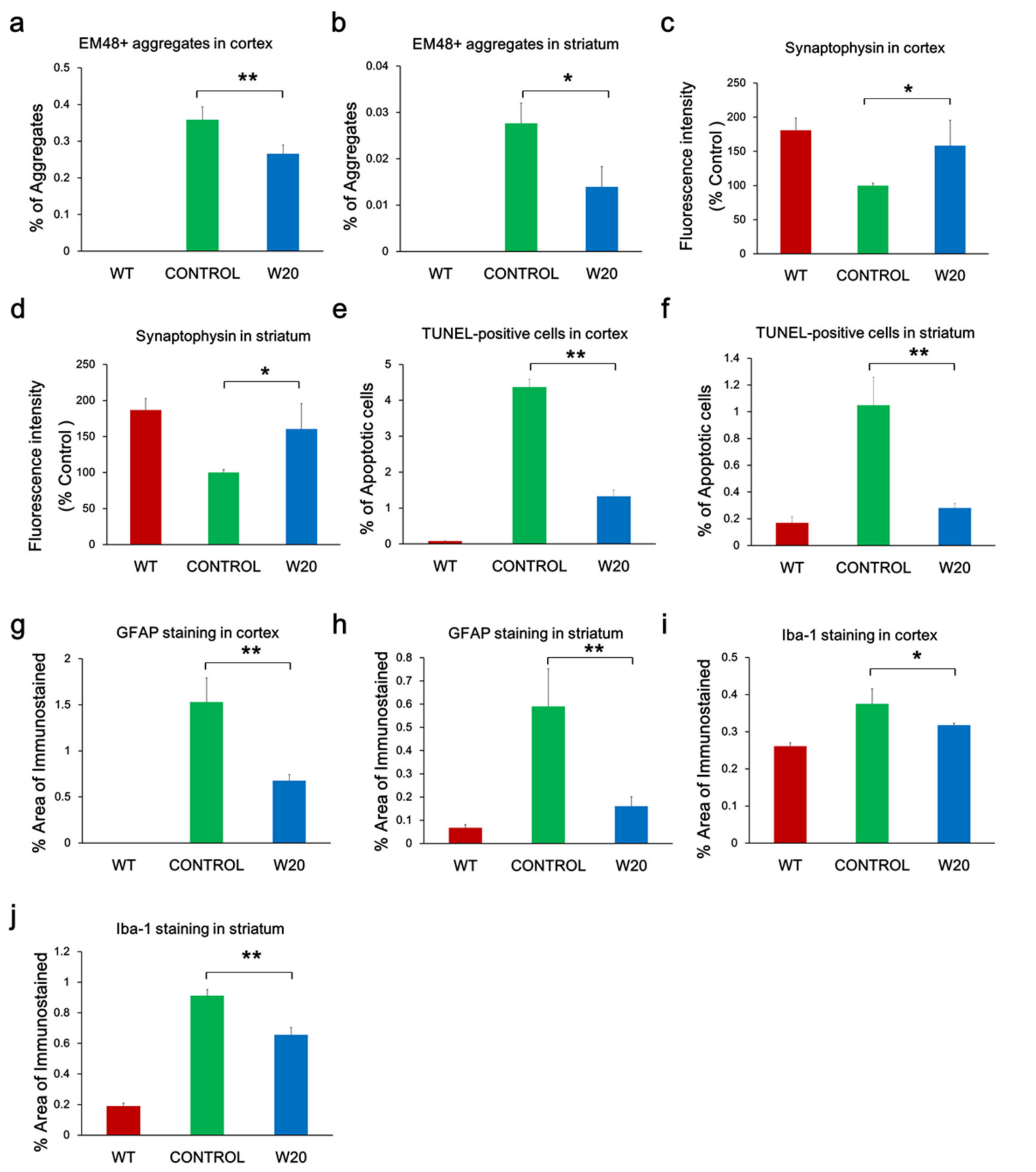

Supplementary Figure 4. W20 reduced neuropathology and neuroinflammation in BACHD mice. EM48 (**a,b**) and synaptophysin (**c,d**) immunostaining, TUNEL analysis (**e,f**), GFAP (**g,h**) and Iba-1 (**i,j**) immunostaining in the cortex and striatum of WT and BACHD mice treated with or without W20 were quantitative analyzed. $n = 6$ mice/group. *$P < 0.05$, **$P < 0.01$, Student's *t*-test. Data represent means ± SD.

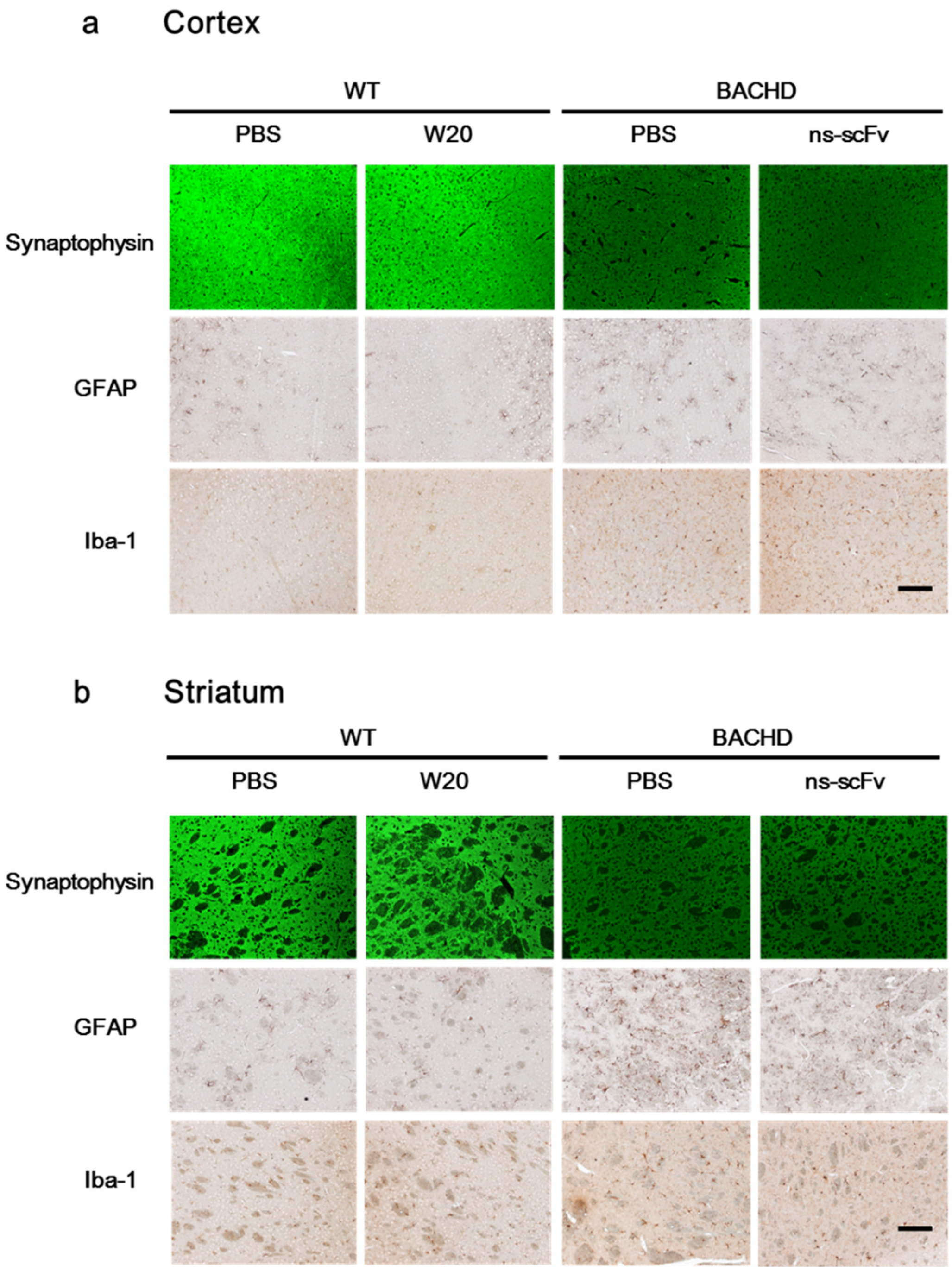

Supplementary Figure 5. Non-specific scFv did not affect the levels of synaptophysin, GFAP and Iba-1 in BACHD mice. Synaptophysin, GFAP and Iba-1 in the cortex (**a**) and striatum (**b**) of WT and BACHD mice were detected with immunohistochemistry. *n* = 6 mice/group. Scale bars, 100 μm.

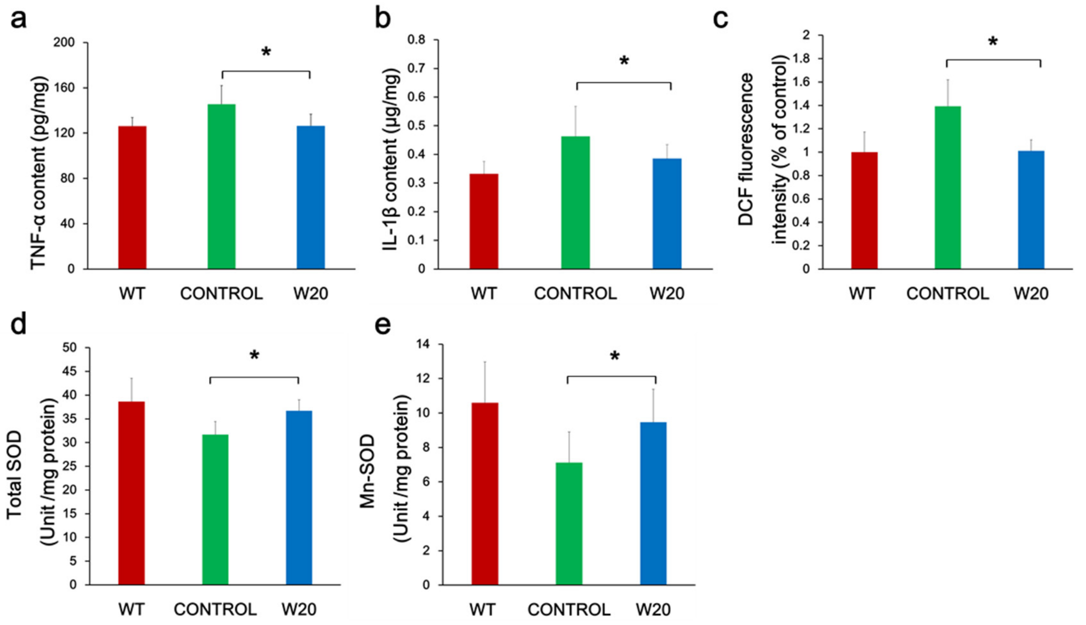

Supplementary Figure 6. W20 decreased proinflammatory cytokine production and attenuated oxidative stress in BACHD mice. Levels of TNF-α (**a**) and IL-1β (**b**) in brain lysates of WT and BACHD mice treated with or without W20 were determined using corresponding ELISA kits. The levels of ROS (**c**), total-SOD (**d**) and Mn-SOD (**e**) in the brain lysates of WT and BACHD mice treated with or without W20 were determined using corresponding commercial kits. n = 6 mice/group. *P < 0.05, Student's t-test. Data represent means ± SD.

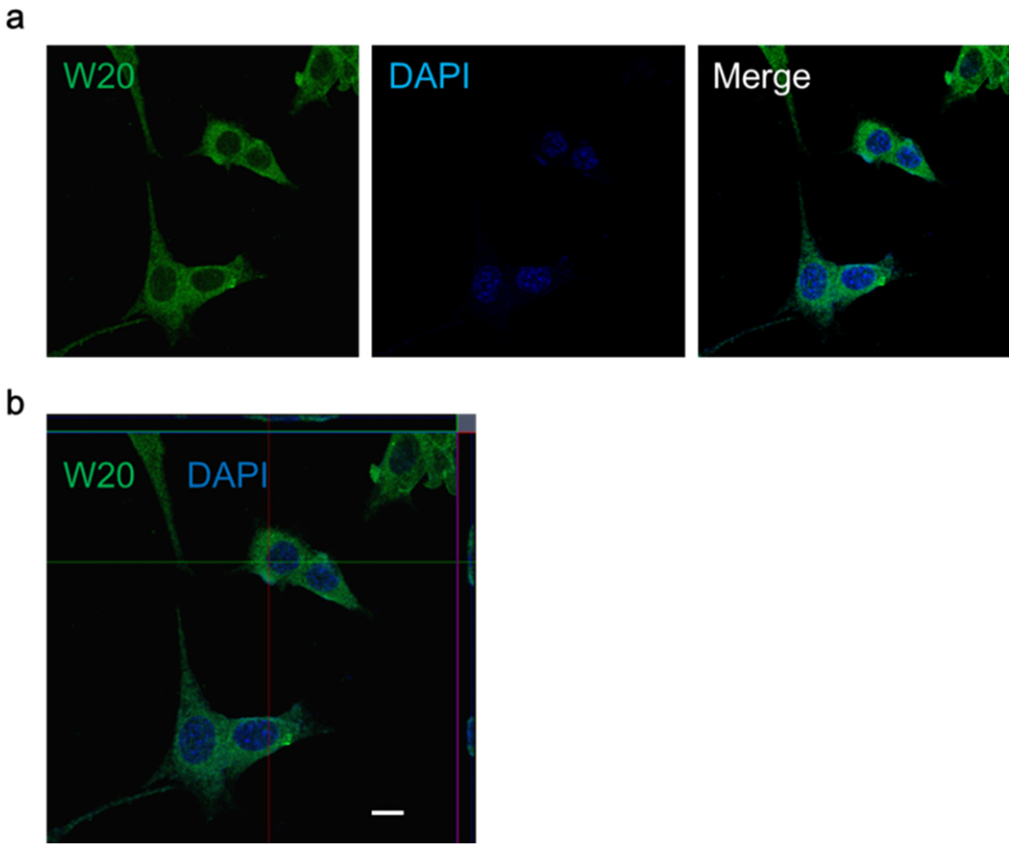

Supplementary Figure 7. W20 was internalized and located in the cytoplasm of HT22 cells. (**a**) HT22 cells were double stained to visualize W20 (green) and DNA (blue), and observed by confocal microscopy. (**b**) Orthogonal view of confocal images with Z-stacks showed cytoplasmic localization of W20. Scale bars, 10μm.

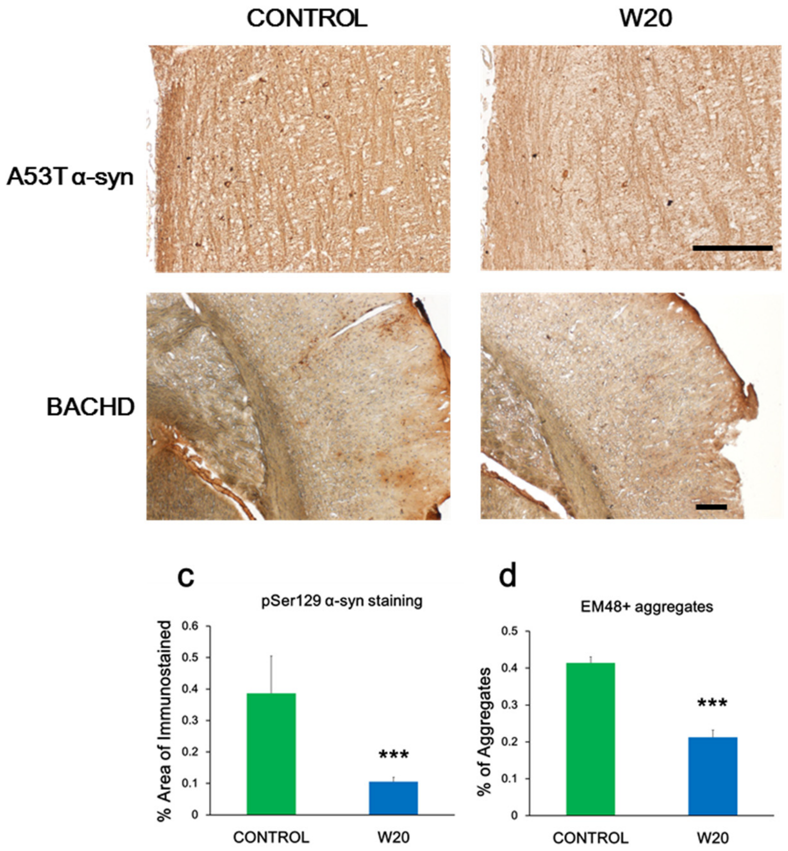

Supplementary Figure 8. W20 disassociated preformed amyloid deposits. The brain slides from A53T α-synuclein (**a**) and BACHD (**b**) were incubated with W20 overnight at 37 °C, and then immunostained by phospho-Ser129-α-synuclein and EM48, respectively. The deposits were quantitatively analyzed (**c, d**). Scale bars, 200μm.
